# TFEB, SIRT1, CARM1, Beclin-1 expression and PITX2 methylation in breast cancer chemoresistance: a retrospective study

**DOI:** 10.1186/s12885-021-08844-y

**Published:** 2021-10-18

**Authors:** Serena Bertozzi, Ambrogio P. Londero, Luigi Viola, Maria Orsaria, Michela Bulfoni, Stefania Marzinotto, Bruna Corradetti, Umberto Baccarani, Daniela Cesselli, Carla Cedolini, Laura Mariuzzi

**Affiliations:** 1 Breast Unit, DAME, University Hospital of Udine, Piazza Santa Maria della Misericordia, 15, 33100 Udine, Italy; 2 Ennergi Research (non-profit organisation), 33050 Lestizza, UD Italy; 3 Clinic of Obstetrics and Gynecology, University Hospital of Udine, Piazza Santa Maria della Misericordia, 15, 33100 Udine, Italy; 4 Department of Radiology & Radiotherapy, University of Campania “Luigi Vanvitelli”, 80100 Naples, Italy; 5 Institute of Pathology, DAME, University Hospital of Udine, 33100 Udine, UD Italy; 6 Department of Nanotechnology, Houston Methodist Hospital, Houston, TX USA; 7 Clinic of Surgery, DAME, University Hospital of Udine, 33100 Udine, UD Italy

**Keywords:** TFEB, SIRT1, CARM1, Beclin-1, PITX2, Breast cancer, Chemoresistance

## Abstract

**Background:**

Breast cancer chemoresistance is attributed to a wide variety of mechanisms, including autophagy. Transcription factor EB (TFEB) has been recently identified and characterized as one major regulator of autophagy and lysosomal genesis.

**Objective:**

This study aims to evaluate the prognostic impact of TFEB and its pathway in breast cancer chemoresistance.

**Methods:**

This retrospective study analyzes the expression of TFEB, CARM1, SIRT1, and Beclin-1 and the methylation of PITX2 in breast carcinoma. A group of breast cancer patients treated with chemotherapy, who relapsed within 12 months from treatment initiation, were compared to a sub-cohort of chemo-treated patients who did not recur within 12 months of follow-up. The expression of TFEB, CARM1, SIRT1, and Belcin-1 was analyzed using immunohistochemistry or RT-PCR on formalin-fixed paraffin-embedded samples. PITX2 methylation was tested with the diagnostic CE-marked kit Therascreen PITX2 RGQ PCR. In the final model, 136 cases of chemo-treated breast cancer were included.

**Results:**

A higher TFEB and Beclin-1 expression correlate with shorter survival in patients with chemo-treated invasive breast cancer (respectively HR 3.46, CI.95 1.27–9.47, *p* < 0.05 and 7.11, CI.95 2.54–19.9). TFEB, CARM1, and SIRT1 are positively correlated with Beclin-1. The protein expression of SIRT1 is significantly associated with TFEB and CARM1 so that a very low SIRT1 expression (lower than the first quartile of the H-score distribution) correlates with a low expression of TFEB and CARM1 and with longer survival. SIRT1 seems to have a lower H-score in the basal-like and HER2-enriched tumors than the luminal subtypes. Beclin-1 and TFEB seem to have a higher H-score in the basal-like and HER2-enriched tumors than the luminal subtypes. PITX2 methylation analysis was feasible only in 65% of the selected samples, but no significant differences between cases and controls were found, and there was also no correlation with the expression of the TFEB pathway.

**Conclusions:**

TFEB, SIRT1, and Beclin-1 seem to have a potential prognostic significance in patients with chemo-treated breast cancer, likely because of their role in the regulation of autophagy. In addition, no correlation between TFEB and PITX2 methylation was found, likely because they perform two different roles within the autophagy process.

**Supplementary Information:**

The online version contains supplementary material available at 10.1186/s12885-021-08844-y.

## Highlights - what this paper adds

• A high TFEB expression correlates with shorter survival in patients with chemo-treated invasive breast cancer;

• A high Beclin-1 expression correlates with shorter survival in patients with chemo-treated invasive breast cancer;

• TFEB, CARM1, and SIRT1 are positively correlated with Beclin-1 expression.

• Low SIRT1 expression correlates with a low expression of TFEB and CARM1 and with longer survival;

• PITX2 methylation analysis was feasible only in 65% of the selected samples, but no significant differences between cases and controls were found;

• No correlation between TFEB and PITX2 methylation was found;

• TFEB, SIRT1, and Beclin-1 seem to have a potential prognostic significance in patients with chemo-treated breast cancer.

## Background

Despite the remarkable progress in both diagnosis and treatment, breast carcinoma continues to be a leading cause of cancer-related death among women worldwide. It is the second biggest killer out of all malignancies in the female gender, after lung and bronchial neoplasia [1, 2]. In particular, metastatic breast carcinoma remains a major challenge for the breast specialist, and unfortunately, its incidence has remained at a fairly consistent level over recent years [1]. Breast cancer comprises multiple subtypes that present different prognostic outcomes and various molecular patterns [3, 4]. The breast cancer molecular subtypes include luminal A, luminal B, luminal HER2, HER2 enriched, and basal-like [5]. The basal-like subtype presents commonly high tumor grading, and adverse outcomes (they do not express estrogen, progestogen receptors, or HER2) [3, 4]. Meanwhile, Luminal A and B show the most favorable outcomes and express hormonal receptors [6].

As in other cancer histotypes, the role of autophagy and chemoresistance in breast cancer is a critical issue. Drug resistance can be attributed to a wide variety of mechanisms. Pre-clinical studies using chemical inhibitors of autophagy or siRNA to downregulate autophagy genes demonstrate the role of autophagy in the chemotherapy sensitization of cancer cells [7–10]. Among the transcription factors that regulate autophagy, the transcription factor EB (TFEB) was identified and characterized in 2009 as the main regulator of autophagy and lysosomal genesis, as well as an element capable of regulating autophagy by coordinating the expression of genes acting at all stages of the autophagy process [11–14]. The co-activator-associated arginine methyltransferase 1 (CARM1) has a transcriptional coactivator role in autophagy and lysosomal genes through TFEB [15]. Furthermore, the CARM1 dependent histone arginine methylation is a fundamental nuclear event in autophagy [15]. A deacetylase involved in epigenetic changes, SIRT1, also influences the expression of autophagy-related genes [16, 17]. SIRT1 is known to be a significant prognostic factor in many cancers [18], and a high SIRT1 expression is known to be an unfavorable prognostic factor in breast cancer [19, 20]. In addition, Beclin-1 is a known marker of autophagy [21, 22].

Paired-like homeodomain transcription factor 2 (PITX2) works in the Wnt signaling pathway by modulating β-catenin, which in turn affects the transcription of cell cycle-associated and proliferation genes, enhancing cell proliferation [23]. The promoter methylation of the PITX2 gene can lead to gene silencing inducing changes in molecular signaling networks, and it was found to be a prognostic biomarker in breast cancer [23]. The kit for evaluating the methylation of PITX2 (a commercially available CE-marked kit: Therascreen PITX2 RGQ PCR) was found to be a reliable prognostic factor for drug resistance, and it was found suitable if applied on formalin-fixed paraffin-embedded tissue samples. In particular, the methylation of PITX2 has been found to predict sensitivity to anthracycline-based chemotherapy in breast cancer [24–27].

This study’s main objective is to assess the prognostic role of TFEB, CARM1, SIRT1, and Beclin-1 in chemo-treated breast carcinoma and compare the TFEB, CARM1, SIRT1, and Beclin-1 expression with the methylation of PITX2 in breast cancer treated with chemotherapy.

## Methods

### Study population

The study population comprises women affected by invasive breast carcinoma, treated by adjuvant or neoadjuvant chemotherapy, and with archived tissue specimens of the primary tumor. Tissue samples (standard archived, formalin-fixed, paraffin-embedded tissue) and clinical data were retrospectively collected at the Institute of Pathology and at the Breast Unit of our center, respectively.

In this study, all breast cancer cases treated by adjuvant or neoadjuvant chemotherapy between January 2002 and December 2016 were included in order to have at least 12 months of follow-up. All cases were included where sufficient tumor tissue was stored in paraffin blocks at the time of surgery. Consequently, all cases of complete pathological response were excluded, as the quantity of tissue from preoperative biopsy did not provide enough material for the current study. Also, the cases of poorly conserved or quantitatively insufficient tissue were excluded. Furthermore, all of the following cases were excluded from the study: those without a documented follow-up, breast cancer patients who did not undergo adjuvant or neoadjuvant treatments, and male patients.

### Study design

This retrospective observational study reviews pathological archives and medical records to identify breast cancer cases treated by adjuvant or neoadjuvant chemotherapy. For the purpose of the study, two different types of sample selection from the original cohort were performed: case-cohort study; and case-control study.

### Case-cohort study

The case-cohort study design model is a sampling methodology to randomly select a sample (called subcohort) from an assembled epidemiologic cohort and to use this subcohort as a comparison group for the selected cases that occur in the cohort (in this particular case, the group of breast cancer patients that relapsed within 12 months of follow up) [28]. This kind of study is particularly suitable for very numerous cohorts, where it is cost-prohibitive to provide a complete follow-up either for disease outcomes or for specific information on the whole cohort [28–30].

Of the 4504 women in the cohort treated for breast pathology, 894 were eligible for the present study because of chemotherapy treatment and fulfilling the required inclusion criteria. The study sample was selected from the latter 894 women. It was composed of a random group of 163 of the 894 eligible women (hereafter called the subcohort) together with all eligible women diagnosed with a breast cancer recurrence within 12 months of treatment initiation. We considered all recurrences (loco-regional recurrences or distant metastases) and adverse events (breast cancer-related death) occurring between the baseline attendance and April 30, 2017.

The final sample included 203 women: 163 belonging to the subcohort and 42 breast cancer patients who experienced recurrence within a 12-month follow-up. We factored in that about 35% of samples could potentially be unavailable for the assessment in the TMA. Power and sample size considerations were based on Cai and Zeng’s approach, and the study had > 80% power to detect HRs of 3.0, assuming alfa = 0.05 [31].

### Case-control study

In the case-control sample selection, all breast cancer patients included in the tissue micro-array were considered. Cases and controls were randomly selected from the patients mentioned above.

The number of the analyzed samples was established from the maximum available resources to test PITX2 methylation. Therefore, the total number of cases was 13, and the total number of controls was similarly 13.

### Clinical information

Information collected included some patient characteristics, such as the age at diagnosis, the body mass index (BMI), any familial history for breast or ovarian carcinoma, the current fertility status (pre-or post-menopausal), any use of estro-progestin therapies (with contraceptive intent in the pre-menopause or as hormone replacement therapy in the post-menopause). Tumor characteristics considered were the histotype, grading, expression of estrogen receptor (ER), progesterone receptor (PR), HER2/neu and Mib1/Ki-67, as well as any presence of multifocality/multicentricity, perivascular invasion (PVI), peritumoral lymphocytic infiltration, nodal extracapsular invasion or bunched axillary nodes [32]). Surgical and non-surgical treatments were also taken into account for data elaboration.

Pathological specimens were routinely assessed following the European guidelines [33, 34]. In particular, samples sized 30 mm or less were wholly sliced and evaluated, whereas specimens sized over 30 mm underwent sampling based on the European guidelines [33, 34].

The World Health Organization criteria were used to determine the histology [35] and nodal status (TNM classification VII ed. AJCC/UICC, 2009) [36]. AFIP and Elston Ellis’s recommendations were considered while assessing the grading in ductal carcinoma in situ and invasive carcinoma, respectively [37, 38]. Peritumoral lymphocytic infiltration, PVI, multifocality/multicentricity, and nodal status were determined as described in previous studies [39, 40].

Estrogen Receptor (ER), Progesterone Receptor (PR), Mib1/Ki-67, and HER2/Neu expression were evaluated by immunohistochemistry. We defined positive ER or PR where positivity was ≥1% in any nuclear staining. In addition, HER2/Neu was defined as overexpressed when staining 3+ or 2+ with FISH amplification and negative if the value was 0, 1+ or 2+ without FISH amplification. Through the combination of ER, PR, HER2/Neu, and Mib1/Ki-67, all invasive breast cancers were classified in the following molecular subtypes as previously described: luminal A, luminal B, luminal HER2, HER2-enriched, and basal-like [39].

### Immunohistochemistry and molecular biology analyses

In this study, the following analyses were performed in both the case-cohort and the case-control subject selection. We analyzed the presence and the quantity of mRNA and the relative protein synthesis in the selected breast cancer tumor samples. In addition (solely for subjects selected for the case-control study), the Therascreen PITX2 RGQ PCR was used to test the PITX2 methylation.

### Real-time PCR (RT-PCR) to quantify mRNA of TFEB, CARM1, and SIRT1

To perform real-time quantitative PCR, the primers were prepared for TFEB, CARM1, and SIRT1 (Supplemental Table 1). Glyceraldehyde-3-phosphate dehydrogenase (GAPDH) was used as a housekeeping protein (Supplemental Table 1). The mRNA was extracted from formalin-fixed paraffin-embedded tumor tissue samples by manually micro-dissecting the tumor area that had been histologically marked by a pathologist and using an RNeasy kit (Qiagen®, Venlo, Netherlands) that was used following the manufacturer’s instructions. RNA quantity and purity were measured using the Qubit 2.0 spectrophotometer (Invitrogen®, Carlsbad, CA), and RNA integrity was quantified using the RIN (RNA integrity number) assessed by Agilent 2100 Bioanalyzer (Agilent Technologies®, Santa Clara, CA). From this m-RNA, employing retrotranscription (SuperScript®, III REV transcript; Life Technologies), cDNA was obtained and quantified. Quantitative real-time PCR analysis was performed in triplicate by three independent experiments, using the LightCycler® 480 (Roche®) and LC SYBR Green I Master (Roche®), according to the manufacturers’ instructions. Quantitative data were collected as cycle threshold (CT) values considering the means of the triplicate runs. Finally, quantitative ΔCT expression values (2^(−ΔCT)^) were calculated.

### Immunohistochemical analysis (IHC)

Along with the RT-PCR analysis, TFEB, CARM1, and SIRT1 protein expressions for all breast cancer cases in our samples were investigated by immunohistochemistry. Beclin-1 was investigated by immunohistochemistry. Cases were evaluated with respect to both staining percentage and intensity.

### Tissue Micro Array (TMA)

The preparation and analysis of the TMA were carried out as previously described [41–46]. Having identified formalin-fixed, paraffin-embedded tumor tissue corresponding with our sample of patients, the hematoxylin-eosin-colored sections were analyzed. Then the tissue core samplings for the TMA were performed, taking care to include neoplastic tissue (two core biopsies per primary tumor). The receiver blocks were assembled. From these, 4-μm cross-sections were obtained, which were stained with hematoxylin-eosin. At a later stage, additional 4-μm cross-sections were obtained to prepare slides for immunohistochemical staining and subsequent analysis.

Immunohistochemical staining was performed according to standard protocol and manufacturer instructions. For antigen retrieval, the slides were heated, after deparaffinization, for 20 min at 98 °C in Target Retrieval Solution (low pH; Dako K8005, Glostrup, DK) with PT-link (Dako), and endogenous peroxidase activity was blocked with H2O2 for 10 min at environmental temperature. Slides were rinsed in PBS and then incubated with the following primary antibodies for 1 h at room temperature: TFEB (OriGene Technologies Inc., diluted 1:100, Rockville, MD, USA); CARM1 (OriGene Technologies Inc., diluted 1:100, Rockville, MD, USA); SIRT1 (OriGene Technologies Inc., diluted 1:200, Rockville, MD, USA); and Beclin-1 (Abcam plc., diluted 1:100, Cambridge, UK). A Dako REAL™ EnVision™ Dako Rabbit/Mouse (Dako, K5007, Glostrup, DK) was used as a second antibody. HRP activity was identified utilizing Dako REAL™ DAB+Chromogen (Dako, K5007, Glostrup, DK) as the substrate for 3 min in accordance with the manufacturer’s instructions. Tissue sections were then counterstained with hematoxylin. Negative controls included sections incubated with non-immune rabbit serum instead of the primary antibody. The immunohistochemical staining was evaluated independently by two pathologists in terms of H-score (the product of the actual percentage of positive-stained cells and intensity score, evaluated as strong 3, moderate 2, and weak 1, giving a possible range of 0–300).

### PITX2 DNA methylation assay

The PITX2 promoter methylation was evaluated for DNA extracted from formalin-fixed paraffin-embedded sections (QIAmp DNA mini kit-Qiagen). Specifically, DNA was extracted from each primary tumor tissue sample kept at room temperature until the extraction time. Therascreen® PITX2 RGQ PCR kit is a methylation-specific PCR (MSP) based on real-time PCR, intended for the quantitative assessment of percent methylation ratio (PMR) in the promoter 2 (P2) of the PITX2 gene and it was validated in primary formalin-fixed paraffin-embedded breast cancer tissue [23, 47, 48]. PMR was calculated according to manufacturer instructions.

Genomic DNA was extracted from the samples using the QIAmp DNA FFPE Tissue Kit (QIAGEN Inc.). DNA was quantified using a Qubit dsDNA analysis kit for QBIT 2.0 fluorimeter (Thermo Fisher Scientific). The bisulfite conversion of the DNA was performed using the EpiTect Plus DNA Bisulfite Kit (QIAGEN Inc.), and the methylated DNA was then purified using the purification module reagents provided by the same kit and quantified at QBIT 2.0. After bisulfite conversion, the PMR of 3 CpG motifs of the PITX2 gene P2 was quantified by MSP using the Therascreen® PITX2 RGQ PCR kit, this containing a quantitative RT-PCR reaction mix, a primer, probes, and positive and negative controls [47].

The quantitative real-time PCR reaction was performed using the Rotor-Gene Q MDx real-time PCR platform (QIAGEN, Inc.) and evaluated by QIAGEN Rotor-Gene AssayManager® (Version 2.1.0) software with Therascreen PITX2 FFPE (C) analysis plugin for analysis and quality control [49].

During all tissue sample analyses, the operators were blinded to the clinical data of the patients. All primary formalin-fixed paraffin-embedded breast cancer tissue samples were coded to ensure blinding of the operator while conducting the PITX2 DNA methylation assay.

### Statistical analysis

Data were analyzed through R (version 3.6.1; R Core Team (2019); R: A language and environment for statistical computing. R Foundation for Statistical Computing, Vienna, Austria; URL https://www.R-project.org/) and considering *p* < 0.05 as significant. The normality of the distribution was tested with the Kolmogorov-Smirnov test. Univariate analysis was performed by the Wilcoxon or t-test in cases of continuous variables, or the Fisher exact test or chi-square test in cases of categorical variables. Univariate and multivariate survival analyses were also performed by Kaplan-Meier curves, Log-rank test, and Cox proportional hazards regression models. OS was considered to be the main outcome. Besides, in the multivariate model, all selected factors and their interactions were accommodated in a single analysis, except when the interaction term was non-significant (in which case we analyzed the non-interaction model). Correlations were tested by Spearman Rho and the relative *p*-value.

## Results

### Population description

After the identification of the forty eligible subjects, the sub-cohort of 163 patients was extrapolated. From this first procedure, it was found that four subjects were duplicated between the cases and the sub-cohort. The blocks for the creation of the TMA were then collected. During the creation of the TMA of the 199 selected samples (including 159 that did not relapse within a 12 month follow up and 40 cases that did), only 109 cases that did not relapse within the year and 27 cases that did were able to be successfully included in the TMA. In the final TMA, 50 cases without relapse within 12 months and 13 relapsed cases had to be excluded, as their associated samples were unusable (i.e., they were used in other studies, there was an insufficient quantity of residual neoplastic tissue, or sample was simply not available).

Table 1 shows a description of the included samples. The median age at breast cancer surgery was 55 years (48–65), and the median BMI was 25 kg/m2 (IQR 22–29). In 99.26 and 8.82% of cases, respectively, adjuvant and neoadjuvant chemotherapy was administered. With regards to the surgical treatment, mastectomy was performed in the majority of cases (68.38%), together with complete axillary lymph node dissection (69.12%).

**Table 1:** Population and tumor characteristics

Age (years)	55 (48–65)
BMI (kg/m^2^)	25 (22–29)
Tobacco smoke	12.5% (12/96)
Family history	42.22% (19/45)
EP use	31.82% (7/22)
Post-menopausal status	71.32% (97/136)
Definitive breast surgery	
Conservative	31.62% (43/136)
Mastectomy	68.38% (93/136)
Axilla surgery: definitive CALND	69.12% (94/136)
Non-surgical therapy	
Neoadiuvant chemoterapy	8.82% (12/136)
Radiotherapy	56.62% (77/136)
vAdiuvant chemoterapy	99.26% (135/136)
Anti-hormonal therapy	74.26% (101/136)
**Histology**	
Invasive ductal carcinoma	80.88% (110/136)
Invasive lobular carcinoma	11.03% (15/136)
Invasive ductal and lobular carcinoma	6.62% (9/136)
Other invasive cancers	1.47% (2/136)
**Molecular types**	
Luminal A	18.38% (25/136)
Luminal B	28.68% (39/136)
Luminal HER2	8.09% (11/136)
HER2 enriched	11.03% (15/136)
Basal-like	13.97% (19/136)
Not described	19.85% (27/136)
**Other tumor caracteristics**	
Mib-1/Ki-67 (median percentage)	30 (10–70)
Mib-1/Ki-67 (> 20%)	55.77% (58/104)
Comedo-like nescrosis	13.24% (18/136)
Multifacality/multicentricity	24.26% (33/136)
Extended in situ component	21.32% (29/136)
Perivascular invasion	40.44% (55/136)
**Lymph node characteristics**	
Micro-metastatic lymph nodes	5.88% (8/136)
Extracapsular invasion	25.74% (35/136)
Axilla lymph node bunching	7.35% (10/136)

Acronyms: *BMI* body mass index, *SLNB* sentinel lymph node biopsy, *CALND* complete

axilla lymph node dissection

Lymph node macrometastases (neoplastic cell aggregates > 2 μm) and micrometastases (neoplastic cell aggregates of 0.2–2 μm) were discovered in 56.62 and 5.88% of cases, respectively. Considering that before 2015 both macro-and micrometastases were considered an absolute indication for complete axillary lymph node dissection and that this study includes patients operated on between 2002 and 2016, it is likely that the great majority of these cases underwent complete axillary lymphadenectomy. The remaining cases who underwent axillary lymph node dissection included patients who were clinically node-positive before neoadjuvant treatment and converted to clinically node-negative afterward (Table 1), as these particular cases are still the subject of worldwide debate, and there is no unanimous consensus about the best management of their axillary lymph nodes.

In Table 1, further tumor characteristics are described. In 80.88% of cases, the tumor histotype was invasive ductal carcinoma, followed by invasive lobular carcinoma in 11.03% of cases. 55.77% of tumors presented a Mib-1/Ki-67 greater than 20, and 24.26% were found to be histologically multifocal or multicentric.

Table 2 reports breast tumor staging. Most tumors were classified as TNM II and III (respectively 36.03 and 24.26%), and tumor grading was classified as G2 in more than half of patients (52.21%).

**Table 2:** TNM staging and tumor grading

T (TNM)	
T1	53.68% (73/136)
T2	36.03% (49/136)
T3	5.88% (8/136)
T4	4.41% (6/136)
N (TNM)	
N0	37.5% (51/136)
N1	36.03% (49/136)
N2	11.76% (16/136)
N3	14.71% (20/136)
TNM stage	
I	34.56% (47/136)
II	36.03% (49/136)
III	24.26% (33/136)
IV	5.15% (7/136)
Tumor grading	
G1	7.35% (10/136)
G2	52.21% (71/136)
G3	40.44% (55/136)

Acronyms: *TNM*tumor node metastasis

### TFEB, CARM1, SIRT1, and Beclin-1 expression

**Fig. 1:**
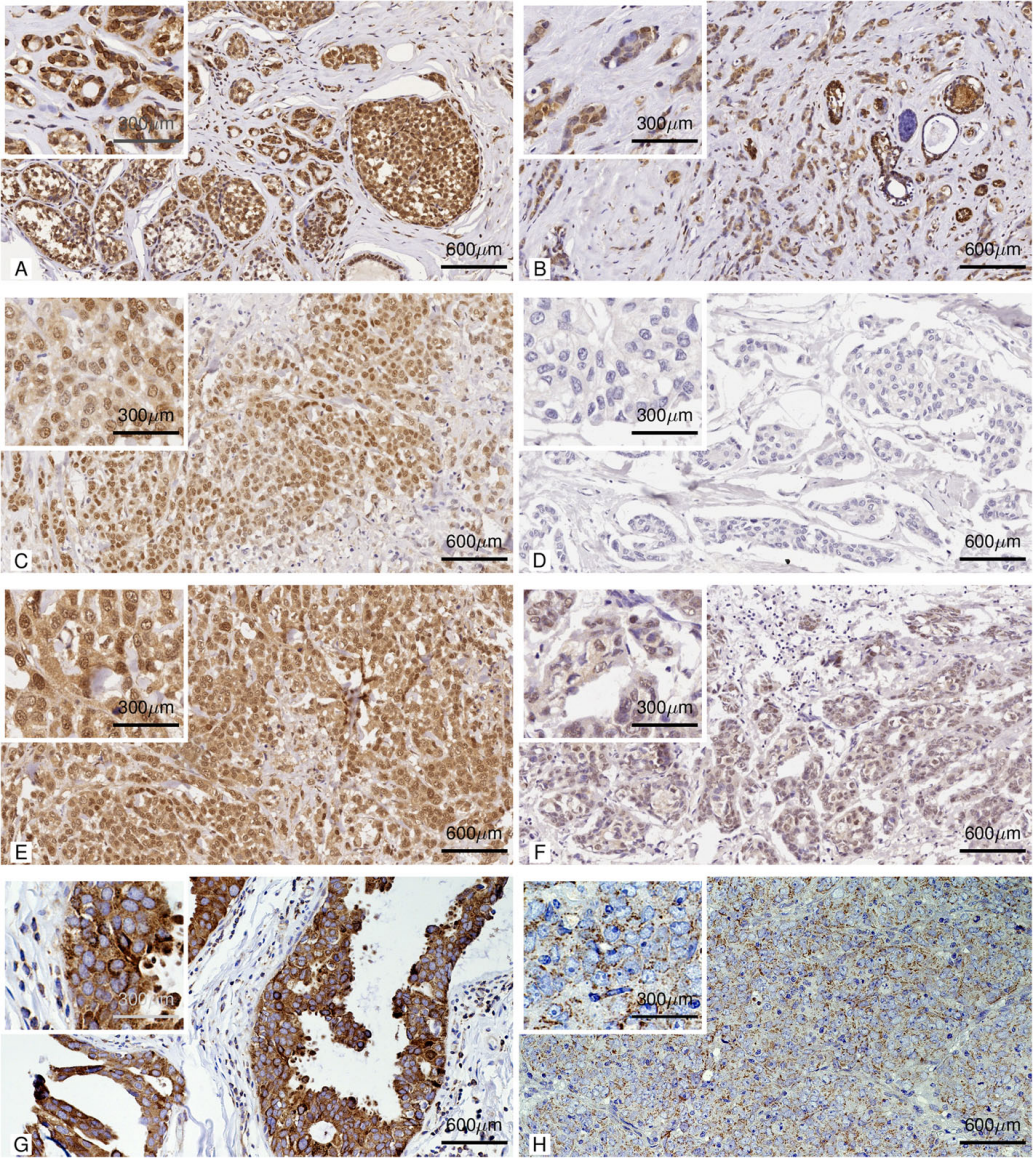
Immunohistochemical staining. **A** Image at 20x (and in the box at 40x) of primary breast cancer tissue TFEB immunohistochemical staining in a patient with breast cancer recurrence within 12 months of follow-up. **B** Image at 20x (and in the box at 40x) of TFEB immunohistochemical staining in a patient’s primary breast cancer tissue without recurrence within 12 months of follow-up. **C** 20x image (and in the 40x square) of CARM1 immunohistochemical staining in primary breast cancer tissue of a woman with recurrent breast cancer within 12 months of follow-up. **D** 20x image (and in the 40x frame) of CARM1 immunohistochemical staining in a primary breast cancer specimen of a patient which did not recure within 12 months of follow-up. **E** Image at 20x (and in the box at 40x) of SIRT1 immunohistochemical staining in a primary breast cancer specimen of a woman with recurrent breast cancer within 12 months of follow-up. **F** Image at 20x (and in the box at 40x) of SIRT1 immunohistochemical staining in a primary breast cancer specimen of a woman without tumor recurrence within 12 months of follow-up. **G** Image at 20x (and in the box at 40x) of Beclin-1 immunohistochemical staining in a primary breast cancer specimen of a woman with recurrent breast cancer within 12 months of follow-up. **H** Image at 20x (and in the box at 40x) of Beclin-1 immunohistochemical staining in a primary breast cancer specimen of a woman without tumor recurrence within 12 months of follow-up

The Median TFEB expression in terms of H-score is 185 (IQR 99–200) with an exclusive nuclear localization (Fig. 1A and B). Median CARM1 expression in terms of H-score is 85 (IQR 25–100) again with a nuclear localization (Fig. 1C and D). The median SIRT1 expression is 190 (IQR 100–200), similarly with a localization in the nucleus (Fig. 1E and F). Finally, The Median Beclin-1 expression in terms of H-score is 90 (IQR 49–162) with an exclusive cytoplasmic localization (Fig. 1G and H).

**Fig. 2:**
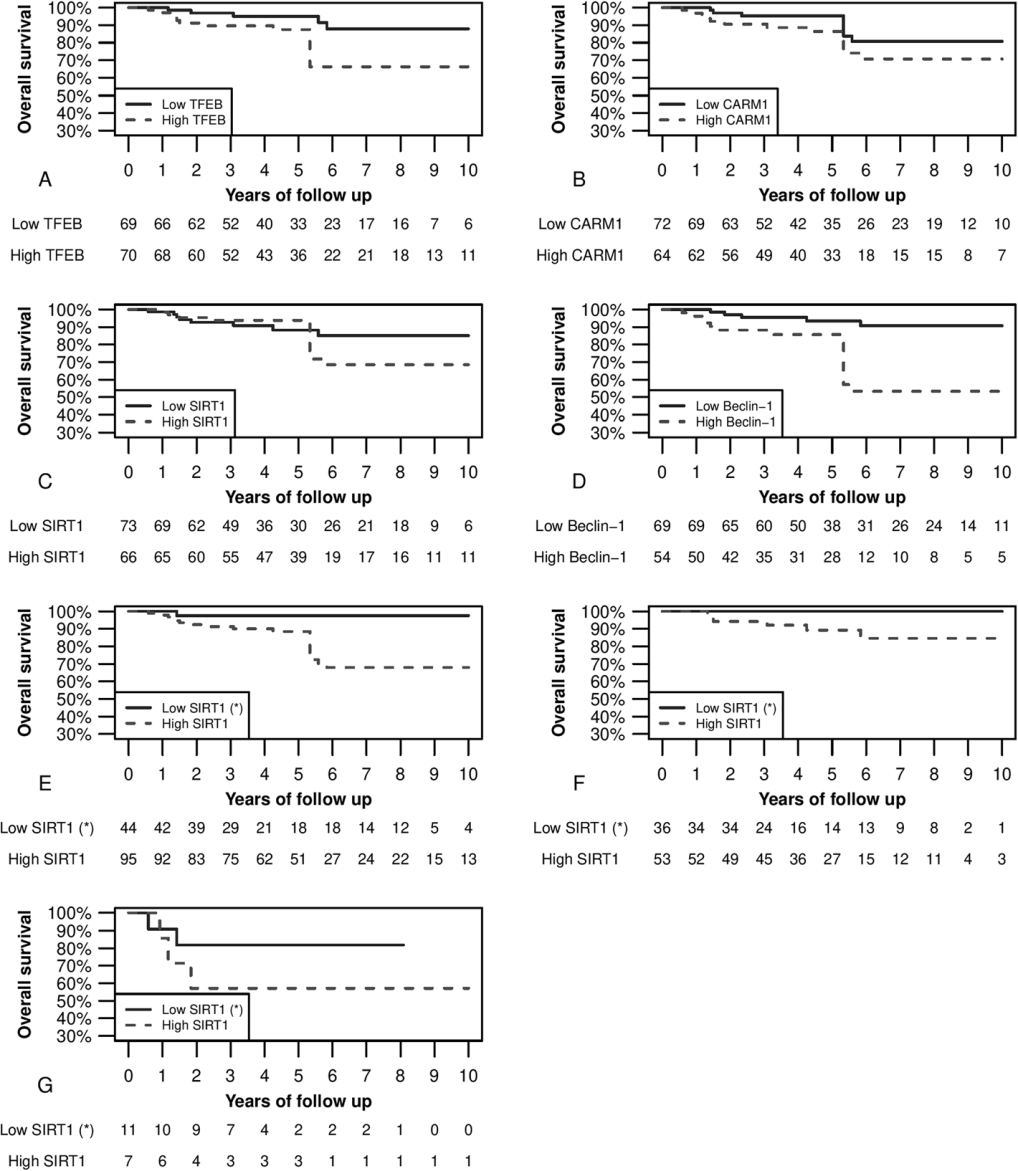
Kaplan-Meier survival curves and *p*-values refers to Log-rank tests. **A** Curve based on TFEB expression (high expression consists in an H-score greater than the distribution median [> 185] and low expression in an H-score lower or equal to the distribution median) (*p* < 0.05). **B** Analysis based on CARM1 expression (i.e., high with an H-score higher than the median distribution [> 85] or low, i.e., with an H-score lower than or equal to the distribution median) (*p* = 0.156). **C** Analysis of survival based on SIRT1 expression (i.e., high with an H-score higher than the median distribution [190] or low, i.e., with an H-score lower than or equal to the distribution median) (*p* = 0.322). **D** Analysis of survival based on the expression of Beclin-1 (i.e., high with an H-score higher than the median distribution [> 90] or low, i.e., with an H-score lower than or equal to the distribution median) (*p* < 0.05). **E** Analysis of survival based on the expression of SIRT1 with a different cut-off (i.e., high with an H-score higher than the first quartile (*) of the distribution [> 100] or low, i.e., with an H-score lower than or equal to the first quartile of the distribution) (*p* < 0.05). **F** Subgroup analysis of survival (luminal A, luminal B, and luminal Her sub-types) based on the expression of SIRT1 (i.e., high with an H-score higher than the first quartile (*) of the distribution [132] or low, i.e., with an H-score lower than or equal to the first quartile of the distribution) (*p* = 0.055). Panel G: Subgroup analysis of survival (Her enriched and Basal-like sub-types) based on the expression of SIRT1 (i.e., high with an H-score higher than the first quartile (*) of the distribution [132] or low, i.e., with an H-score lower than or equal to the first quartile of the distribution) (*p* = 0.294)

Figure 2A highlights the analysis of survival based on the expression of TFEB. In particular, a higher immunohistochemical expression of TFEB protein correlates with significantly shorter survival (*p* < 0.05). This difference is also present in the Cox regression analysis with an HR of 3.46 (IC.95 1.27–9.47) (*p* < 0.05). Furthermore, in the multivariate analysis, this difference is shown to be independent of neoadjuvant chemotherapy (Table 3).

**Table 3:** Survival analysis using the univariate and multivariate Cox proportional risk regression model (**), for the analyzes a H-score was considered as high, the one above the median of the distribution

	HR (IC95%)	*p*	HR (IC95%) (**)	*p*(**)
TFEB H-score > 185 (*)	3.25 (1.19–8.86)	< 0.05	3.46 (1.27–9.47)	< 0.05
Beclin-1 H-score > 90 (*)	5.36 (1.96–14.68)	< 0.05	7.11 (2.54–19.9)	< 0.05
Neoadiuvant chemotherapy	7.29 (3.1–17.1)	< 0.05	7.88 (3.26–19.05)	< 0.05

(*) H-score greater than the distribution median

(**) Multivariate model

Figure 2B shows the association between a high expression of CARM1 and shorter survival, although this correlation is not statistically significant. Meanwhile, Fig. 2C shows that an H-score of SIRT1 higher than the distribution median is not associated with significant differences in terms of survival.

Figure 2D shows that a higher immunohistochemical expression of Beclin-1 correlates with significantly shorter survival (*p* < 0.05). This difference is also present in the Cox regression analysis with an HR of 5.36 (IC.95 1.96–14.68) (*p* < 0.05). Moreover, in the multivariate analysis, this difference is shown to be independent of neoadjuvant chemotherapy (Table 3).

### Correlation among immunohistochemical expression of TFEB, CARM1, SIRT1, and Beclin-1

In Supplemental Table 2 and Supplemental Fig. 1, it is possible to observe the correlation among the four analyzed proteins’ expressions. In particular, the immunohistochemical protein expression of SIRT1 correlates directly proportional and significantly with the expression of TFEB, CARM1, and Beclin-1. In addition, the immunohistochemical expression of TFEB also significantly correlates with that of CARM1 and Beclin-1. Finally, the immunohistochemical expression of CARM1 also significantly correlates with that of Beclin-1.

The graph in Supplemental Fig. 1 also shows that a SIRT1 expression within the first quartile of the H-score distribution corresponds to a low expression of CARM1 and TFEB. We consequently decided to evaluate whether survival in this particular subgroup was actually longer. Accordingly, Fig. 2E shows better survival (*p* < 0.05) in the case of SIRT1 expression under the first quartile of the H-score distribution.

### Tumor molecular subtypes and TFEB, CARM1, SIRT1, and Beclin-1 expression

Supplemental Table 3 demonstrates no significant modification to either the CARM1 or SIRT1 result when considering the different tumor molecular subtypes separately. Although not significant, it is remarkable that the H-score of SIRT1 is lower in the basal-like and in the HER2-enriched subtypes than the luminal A, luminal B, and luminal HER2 ones.

Therefore, in Fig. 2F, the Kaplan-Meier survival analysis based on SIRT1 expression was redesigned to include only the luminal A, luminal B, and luminal HER2 molecular subtypes. Under this analysis, no more adverse events were recorded where we observed low SIRT1 expression (*p* = 0.055).

Furthermore, Fig. 2G shows that, also in the basal-like and in the HER2-enriched subtypes, separately, a low SIRT1 expression is associated with a better prognosis, although not in a statistically significant way.

We also redesigned the correlations between the three molecules in Supplemental Table 2, considering the tumor molecular subtypes. In particular, considering only the basal-like subtype, the correlation between CARM1 and SIRT1 is maintained while the TFEB and Beclin-1 correlations with SIRT1 and CARM1 lose their statistical significance. Finally, considering only the luminal variants (luminal A, luminal B, and luminal HER2), the directly proportional and significant correlations of SIRT1 with TFEB and CARM1 are confirmed as well as the correlation between TFEB and Beclin-1 (Supplemental Table 2).

### TFEB, SIRT1, and CRAM1 expression in RT-PCR

The mRNA quantification was only possible in samples with high H-score and low paraffin storage duration. In particular, for CARM1, which was the least expressed protein in immunohistochemistry, no mRNA was detected in the analyzed samples. As regards TFEB (rho = 0.50) and SIRT1 (rho = 0.15), a directly proportional correlation between immunohistochemical score (assessed as H-score) and mRNA expression (assessed as a delta-CT expression) was found, although these correlations are not statistically significant.

### PITX2 methylation (case-control study)

The test failed in 9 samples (35%) (5 cases and 4 controls) of the total 26 tested samples. PMR was found to be > 12 in the 37.50% of cases (3/8) and in 55.56% of controls (5/9) (*p* = 0.637).

Supplemental Table 4 shows the characteristics of the patients in which the test was successful (8 cases and 9 controls), and no statistically significant differences were found. Although not statistically significant, the age of patients in cases with recurrence within the first year of follow-up was greater than in controls, as well as the number of mastectomies, which, however, could be somehow linked to the increased age of patients.

Supplemental Table 4 also shows the tumor characteristics, where again no statistically significant differences were observed - except for a higher prevalence of basal-like tumor subtypes in the case of early recurrences. Supplemental Table 5 shows the tumor stages, and, here again, there were no statistically significant differences between the two considered groups.

In Supplemental Table 6, the differences in the immunohistochemical expression of TFEB, CARM1, and SIRT1 are reported, as well as the PMR value of the PITX2 methylation. Compared with controls, TFEB, SIRT1, and CARM1 expression levels were significantly higher where relapse occurred within 12 months (*p* < 0.05), while no statistically significant differences were observed as regards the PITX2 PMR.

For this same group of patients, considering the correlations between PITX2 methylation and immunohistochemical expression of TFEB, CARM1, and SIRT1, PITX2 methylation does not correlate with the immunohistochemical expression of the rest of the TFEB pathway.

## Discussion

From this analysis of the role of the TFEB pathway in breast cancer chemoresistance, we reveal evidence that the increased expressions of TFEB and Beclin-1 are associated with a shorter survival period in patients suffering from invasive breast cancer who undergo chemotherapy. Our data also demonstrate a significant and directly proportional correlation of SIRT1 expression with TFEB and CARM1 expression, so that a very low expression of SIRT1 (lower than the first quartile of the H-score distribution) shows an associated low expression of TFEB and CARM1, and consequently with a longer duration of survival. Furthermore, in the basal-like and HER2-enriched breast cancer subtypes, SIRT1 seems to have a lower H-score than in the luminal molecular subtypes. Meanwhile, in the basal-like and HER2-enriched breast cancer subtypes TFEB and Beclin-1 seem to have a higher H-score than in the luminal molecular subtypes. As far as PITX2 methylation analysis is concerned, this was feasible only in 65% of the selected cases (of the case-control study). Furthermore, the methylation of PITX2 did not demonstrate any significant differences between the cases and the controls and did not indicate any correlation with the expression of the TFEB pathway analyzed by immunohistochemistry.

The ability to predict the response to anti-blastic therapies in breast carcinoma is of fundamental importance due to the significant prevalence of this disease in females of all ages. Furthermore, despite the effectiveness of screening with improved early diagnosis and the progressive advance of systemic therapies, no reduction has been observed in the prevalence of metastatic breast cancer at the time of diagnosis [1, 5, 50]. In fact, its prevalence does not depend for the most part on the average size of tumors, which today are frequently smaller than in the past, but depends above all on the intrinsic immunophenotypical characteristics of the malignancy. Specifically, tiny tumors with aggressive behavior are far more likely to develop distant metastases than large tumors with limited proliferation [51].

With regard to tumors with aggressive biological behavior, nowadays, chemotherapy remains the most effective tool to reduce the risk of distant metastases. Prognostic factors indicative of unfavorable outcomes, but also predictive of risk reduction in patients undergoing chemotherapy, are tumor size, loco-regional lymph node involvement, tumor histotype, hormone receptor expression, HER2/neu overexpression, Mib-1/Ki-67 proliferation index, tumor grade, perivascular or perineural invasion, and the young age of the patient [5, 32, 34, 52–54]. However, there is still no clarity about the factors which predict tumor chemoresistance. If these factors were available, we would be better equipped to create or design novel drugs to be added to chemotherapy regimens to improve their efficiency.

As for other malignancies, autophagy seems to have a fundamental function in breast cancer resistance to chemotherapy as well as hormonal therapy [7, 9]. In the current literature, it has already been demonstrated that TFEB, SIRT1, CARM1, Beclin-1, and PITX2 play a role of some sort in the autophagy process. However, data explicitly related to breast cancer is extremely limited, especially regarding TFEB. Regarding SIRT1, there are reports which describe a correlation between its increased expression and a poor prognosis in breast cancer [19, 20]. Our study likewise notes an association between SIRT1 expression levels above the first quartile and a diminished survival, and our data confirm a likely association of reduced expression of SIRT1 with the basal-like molecular subtype [55]. Since the correlation between SIRT1 and TFEB disappears when we look exclusively at the basal-like breast cancer subtype, our data actually suggests a different role of SIRT1 in the luminal molecular subtypes compared to that in the basal-like ones, although in both cases, a low expression of SIRT1 is predictive of a better prognosis.

Both TFEB and CARM1 have been seen to play an important role in the onset and regulation of autophagy [15–17]. However, at the moment, only one article demonstrates a correlation between an increased TFEB expression in early breast cancer and a poorer prognosis [56]. Our results confirm that an increased immunohistochemical TFEB expression correlates with a less favorable prognosis in women affected by breast cancer and treated with chemotherapy. Furthermore, our study population extends beyond early breast cancers to encompass many locally advanced tumors, which more commonly represent an indication for chemotherapy. Regarding CARM1, its increased expression corresponds with a less favorable prognosis in breast cancer [6, 57, 58] and with HER2-positive tumors [6, 57]. Our study similarly correlates a higher expression level of CARM1 with a poorer prognosis but did not observe differences concerning the expression of CARM1 among the different molecular subtypes. Again, this may be explained by a potential selection bias, as our population includes, on average, more advanced stages than previous studies.

Several studies have previously assessed the prognostic value of Beclin-1 in breast cancer, showing conflicting results. Dong and coworkers found cancers positively expressing Beclin-1 having a favorable prognosis [59]. However, they limited the analysis to ER-positive and HER2-negative cancers, and only a part of these cases was eligible to be treated with chemotherapy [59]. Won and coworkers found no correlations between Beclin-1 expression and survival [60], as well Kim and coworkers found no correlation between Beclin-1 expression and survival in triple-negative breast cancers [61]. However, the last two studies had an average follow-up below 65 months, and it is known that the majority of recurrences can occur within the first 72 months of follow-up, also considering low-risk breast cancer [53, 60, 61]. Furthermore, Liu and coworkers found an increased expression of Beclin-1 in the tamoxifen-resistant breast cancer cell line [22]. In a cohort of high-risk patients treated with chemotherapy, we found that high Beclin-1 immunohistochemical expression was associated with an unfavorable prognosis.

In this study, we also evaluate the correlations among the three considered proteins. In particular, a directly proportional and statistically significant correlation is found among SIRT1, CARM1, TFEB, and Beclin-1. Previous studies demonstrate that TFEB and CARM1 act as effectors of the autophagy process, and their significant correlation in our results confirms this [15]. Besides, SIRT1, CARM1, and TFEB positively correlated with Beclin-1, a known marker of the autophagy process [21]. Even by selecting only the luminal molecular subtypes, it emerges that the rho coefficient of the correlation between TFEB and CARM1 approaches significance (Supplemental Table 2). On the other hand, this correlation is lost in the case of the basal-like subtype, so that in the breast cancer luminal subtypes, we can deduce that both TFEB and CARM1 act in the same pathway, while this may not be true in the case of the basal-like molecular subtype [15]. The correlation between SIRT1 and CARM1 can be explained by the fact that CARM1, through the methylation of HuR, stabilizes the SIRT1 mRNA and hence promotes its production [62, 63]. Finally, while in the basal-like subtype, both TFEB and CARM1 maintain their prognostic value, the correlation of TFEB with SIRT1 and CARM1 expression loses statistical significance. A possible explanation is that TFEB and CRAM1 may act in two different ways, which at the moment cannot be completely discerned from the data present in the current study.

Considering the case-control study, the prognostic value of TFEB and its pathway in chemo-treated patients is confirmed and probably associated with its function in the autophagy mechanism. In fact, PITX2 also seems to play a role in the autophagy process. Indeed, PITX2 regulates DIRAS3 in lung cancer [64], and the re-expression of DIRAS3 promotes autophagy in breast cancer, thus enhancing the inhibitory effect of paclitaxel on breast tumor cells [65]. Although PITX2 and TFEB both have a crucial function in the autophagy process, their role seems to be different. Our data, in fact, show a significant difference regarding the TFEB pathway but no difference as regards the methylation of PITX2 between cases and controls. Furthermore, our data show no correlation between the methylation of PITX2 and the protein expression of the TFEB pathway, which would suggest the independence of these two prognostic markers.

Currently, adjuvant chemotherapeutic regimens are mostly based on the administration of anthracyclines and taxanes and, for this reason, our study focuses on the evaluation of PITX2, which is a molecule that can specifically influence anthracycline resistance [23–27, 66]. Considering that all patients of our case-control study were treated with polychemotherapy, including anthracyclines, it should be emphasized that avoiding the side effects of anthracyclines when there is evidence of their ineffectiveness could be a valuable step in improving the quality of care in this group of patients.

This study’s major limitations are its retrospective design and the limitations linked to perform an analysis on mRNA and DNA of formalin-fixed paraffin-embedded tissues that have been historically collected and stored for many years. At the same time, it is a crucial advantage to have an extended follow-up that allows a broader perspective on prognosis. Despite its limits, this study shows some significant data of great interest. In fact, an increased TFEB expression in terms of H-score appears to have a marked ability to identify patients with shorter durations of survival. In addition, it should be pointed out among the advantages of this study that the cases were treated uniformly by the same team according to the most up-to-date guidelines.

## Conclusions

Our preliminary data demonstrate a potential prognostic value of TFEB, SIRT1, and Beclin-1, likely for their role within autophagy regulation in patients affected by breast cancer and treated with anti-blastic therapy. As chemotherapy resistance still represents one of the breast specialist’s major concerns, our encouraging data show that the potential exists to discover new treatments to overcome intrinsic or acquired drug resistance in breast carcinoma and, consequently, improve the prognosis of our patients.

## Supplementary Information

**Additional file 1 **: **Supplemental Fig. 1**. The plot shows the correlation analysis between the immunohistochemical protein expression evaluated as the H-score of the four proteins analyzed. Correlations evaluated by the Spearman test.

**Additional file 2 **: **Supplemental Table 1**. RT-PCR primers of TFEB, CARM1, SIRT1, and GAPDH. **Supplemental Table 2**. Analysis of the correlation between the immunohistochemical protein expression evaluated in H-score of the four proteins analyzed. Correlations evaluated by the Spearman test. **Supplemental Table 3**. Analysis of the expression of the considered proteins among the various molecular subtypes where the data is present. The values reported are median and range of interquartiles (IQR) while p refers to the Wilcoxon test. **Supplemental Table 4**. Description of the population and tumor characteristics divided into the two groups (cases who recurred within 12 months from treatment initiation and controls who did not recur). **Supplemental Table 5**. Tumor staging divided into the two groups (cases who recurred within 12 months from treatment initiation and controls who did not recur). **Supplemental Table 6**. PITX2 (PMR) and immunoistochemical expression of TFEB, CARM1, SIRT1, and Beclin-1 divided into the two groups (cases who recurred within 12 months from treatment initiation and controls who did not recur).

## Data Availability

The data that support the findings of this study are available, but restrictions apply to the availability of these data, which was used under license for the current study, and so are not publicly available. Data are however available from the authors upon reasonable request and with permission of the Internal Review Board.

## Abbreviations

BMI: body mass index
CARM1: co-activator-associated arginine methyltransferase 1
CI.95: 95% confidence interval
CT: cycle threshold
ER: estrogen receptor
FFPE: formalin-fixed, paraffin-embedded
GAPDH: glyceraldehyde-3-phosphate dehydrogenase
IHC: Immunohistochemical analysis
IQR: interquartile range
MSP: methylation specific PCR
P2: promotor 2
PITX2: Pituitary homeobox 2
PMR: percent methylation ratio
PR: progesteron receptor
PVI: peri-vascular invasion
RT-PCR: Real time PCR
SIRT1: sirtuin-1
TFEB: Transcription factor EB
TMA: Tissue Micro Array
