# Supplementary figures and images for: TFEB, SIRT1, CARM1, Beclin-1 expression and PITX2 methylation in breast cancer chemoresistance: a retrospective study
Source: BMC Cancer. 2021 Oct 18;21:1118. doi: 10.1186/s12885-021-08844-y (PMC8524961; doi:10.1186/s12885-021-08844-y)

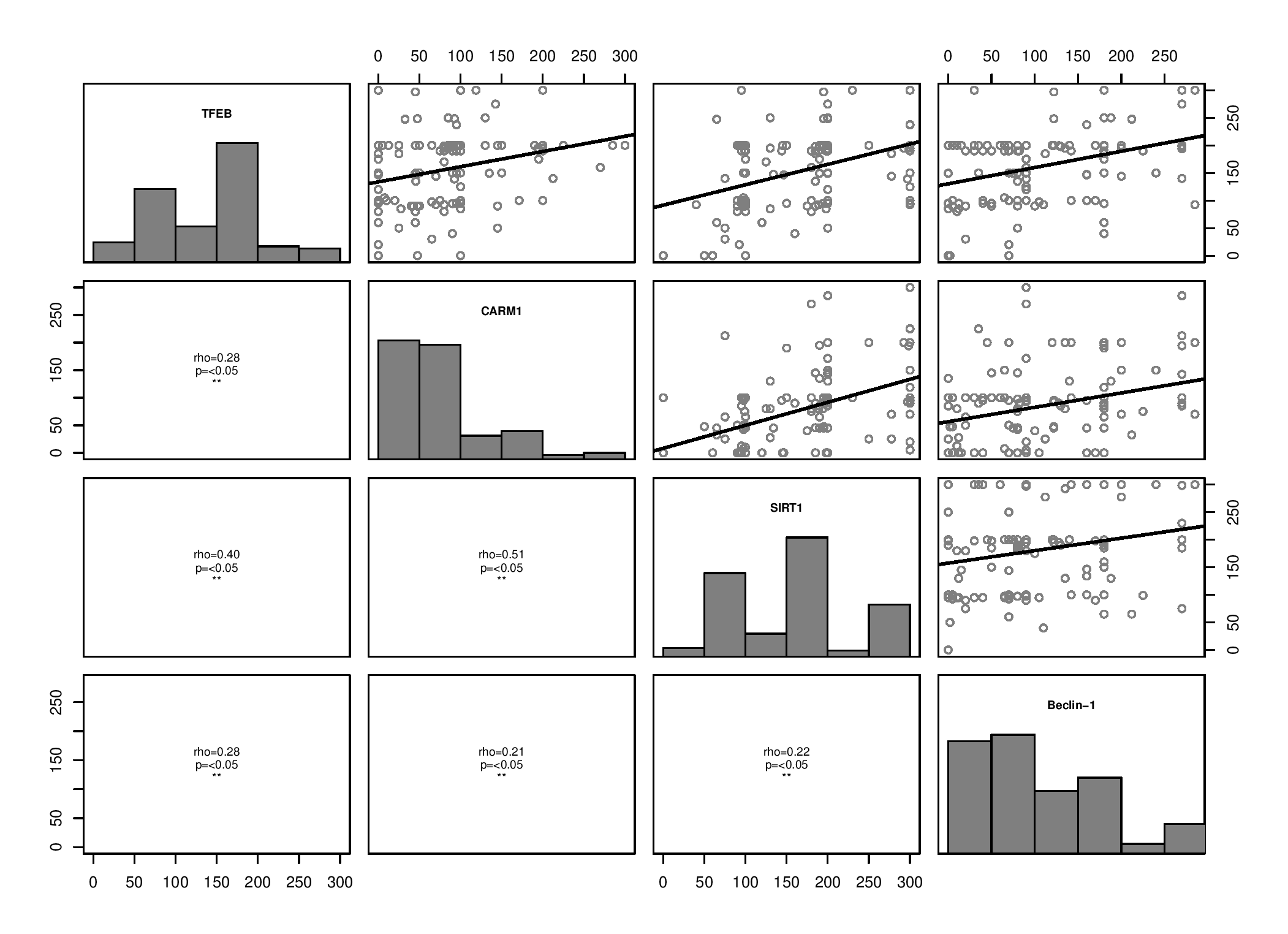

Supplement: Supplementary file 1 — Additional file 1 : Supplemental Fig. 1. The plot shows the correlation analysis between the immunohistochemical protein expression evaluated as the H-score of the four proteins analyzed. Correlations evaluated by the Spearman test. [file 12885_2021_8844_MOESM1_ESM.tif]
