# Supplementary material for: TFEB, SIRT1, CARM1, Beclin-1 expression and PITX2 methylation in breast cancer chemoresistance: a retrospective study
Source: BMC Cancer. 2021 Oct 18;21:1118. doi: 10.1186/s12885-021-08844-y (PMC8524961; doi:10.1186/s12885-021-08844-y)
Supplement: Supplementary file 2 [file 12885_2021_8844_MOESM2_ESM.docx]

Supplemental Table 1- RT-PCR primers of TFEB, CARM1, SIRT1, and GAPDH.

| TFEB | Sequence |
| --- | --- |
| Forward: | TGATCCCCAAGGCCAATGAC |
| Reverse: | TCCCTGGACTTTTGCAGGTC |
| CARM1 |  |
| Forward: | AGCCGGATCTAAGATGGCAG |
| Reverse: | GGGAACACCGACACGGTA |
| SIRT1 |  |
| Forward: | TGCTGGCCTAATAGAGTGGC |
| Reverse: | ACCTCAGCGCCATGGAAAAT |

Suplemental Table 2- Analysis of the correlation between the immunohistochemical protein expression evaluated in H-score of the four proteins analyzed. Correlations evaluated by the Spearman test.

| Correlation | All samples | Basal-like subtype | Luminal A, luminal B and luminal Her subtypes |
| --- | --- | --- | --- |
| TFEB - CARM1 | rho = 0.28, p<0.05 | rho = 0.08, p=0.749 | rho = 0.26, p=0.120 |
| TFEB - SIRT1 | rho = 0.40, p<0.05 | rho = 0.16, p=0.537 | rho = 0.48, p<0.05 |
| TFEB - Beclin-1 | rho=0.28, p<0.05 | rho = 0.31, p=0.217 | rho = 0.33, p<0.05 |
| CARM1 - SIRT1 | rho = 0.51, p<0.05 | rho = 0.69, p<0.05 | rho = 0.42, p<0.05 |
| CARM1 - Beclin-1 | rho=0.21, p<0.05 | rho = 0.13, p=0.600 | rho = -0.047, p=0.797 |
| SIRT1 - Beclin-1 | rho=0.22, p<0.05 | rho = 0.36, p=0.146 | rho = 0.22, p=0.202 |

Supplemental Table 3- Analysis of the expression of the considered proteins among the various molecular subtypes where the data is present. The values reported are median and range of interquartiles (IQR) while p refers to the Wilcoxon test.

|  | Luminal A (24) | Luminal B (38) | Luminal Her (10) | Her enriched (14) | Basal-like (18) | p |
| --- | --- | --- | --- | --- | --- | --- |
| CARM1 H-score | 72.50 (18.75-100.00) | 87.50 (18.75-100.00) | 75.00 (41.38-141.25) | 90.00 (12.50-160.94) | 95.00 (35.62-144.38) | NS |
| SIRT1 H-score | 187.50 (100.00-200.00) | 189.50 (100.00-200.00) | 180.00 (100.00-200.00) | 157.50 (100.00-198.75) | 139.00 (97.88-198.50) | NS |
| TFEB H-score | 150.00 (90.00-196.00) | 112.50 (95.00-193.00) | 142.50 (91.25-200.00) | 192.50 (171.25-199.88) | 190.00 (148.12-200.00) | 6,7 |
| Beclin-1 | 70.00 (35.00-180.00) | 70.00 (20.00-135.00) | 100.00 (80.00-140.00) | 90.00 (31.25-146.25) | 160.00 (82.50-180.00) | 7 |

Statistically significant differences (p <0.05): (1) Luminal A vs Luminal B; (2) Luminal A vs

Luminal Her; (3) Luminal A vs Her enriched; (4) Luminal A vs Basal-like; (5) Luminal B vs

Luminal Her; (6) Luminal B vs Her enriched; (7) Luminal B vs Basal-like; (8) Luminal Her vs

Her enriched; (9) Luminal Her vs Basal-like; (10) Her enriched vs Basal-like. NS = non significant

differences.

Supplemental Table 4- Description of the population and tumor characteristics divided into the two groups (cases who recurred within 12 months from treatment initiation and controls who did not recur).

|  | Recurrence within 12 months (8) | No recurrence (9) | p |
| --- | --- | --- | --- |
| Age (years) | 75.00 (64.00-83.25) | 53.00 (44.00-67.00) | 0.112 |
| BMI (kg/m²) | 25.65 (22.57-26.85) | 23.50 (22.00-25.50) | 0.630 |
| Definitive breast surgery |  |  |  |
| Conservative | 0.00% (0/8) | 44.44% (4/9) | 0.082 |
| Mastectomy | 100.00% (8/8) | 55.56% (5/9) | 0.082 |
| Definitive axilla surgery |  |  |  |
| SLNB | 12.50% (1/8) | 44.44% (4/9) | 0.150 |
| CALND | 87.50% (7/8) | 55.56% (5/9) | 0.150 |
| Neoadjuvant chemotherapy | 37.50% (3/8) | 11.11% (1/9) | 0.200 |
| Histology |  |  |  |
| Invasive ductal carcinoma | 87.50% (7/8) | 77.78% (7/9) | 0.600 |
| Invasive ductal and lobular carcinoma | 12.50% (1/8) | 22.22% (2/9) | 0.600 |
| Molecular types |  |  |  |
| Luminal A | 25.00% (2/8) | 22.22% (2/9) | 1.000 |
| Luminal B | 12.50% (1/8) | 55.56% (5/9) | 0.131 |
| Luminal Her | 0.00% (0/8) | 22.22% (2/9) | 0.471 |
| Her enriched | 12.50% (1/8) | 0.00% (0/9) | 0.471 |
| Basal-like | 50.00% (4/8) | 0.00% (0/9) | <0.05 |
| Other tumor caracteristics |  |  |  |
| Mib-1/Ki-67 (>20%) | 71.43% (5/7) | 50.00% (4/8) | 0.398 |
| Comedo-like nescrosis | 0.00% (0/8) | 11.11% (1/9) | 0.331 |
| Multifacality/multicentricity | 0.00% (0/8) | 22.22% (2/9) | 0.156 |
| Perivascular invasion | 25.00% (2/8) | 44.44% (4/9) | 0.402 |
| Lymph node characteristics |  |  |  |
| Micrometastatic lymph nodes | 0.00% (0/8) | 11.11% (1/9) | 0.331 |
| Extracapsular invasion | 37.50% (3/8) | 0.00% (0/9) | 0.082 |

Acronyms: BMI = body mass index; SLNB = sentinel lymph node biopsy; CALND = complete axillary lymph node dissection.

Supplemental Table 5- Tumor staging divided into the two groups (cases who recurred within 12 months from treatment initiation and controls who did not recur).

|  | Recurrence within 12 months (8) | No recurrence (9) | p |
| --- | --- | --- | --- |
| Tumor size |  |  |  |
| T1 | 12.50% (1/8) | 44.44% (4/9) | 0.294 |
| T2 | 37.50% (3/8) | 55.56% (5/9) | 0.637 |
| T3 | 12.50% (1/8) | 0.00% (0/9) | 0.471 |
| T4 | 37.50% (3/8) | 0.00% (0/9) | 0.082 |
| Nodal involvement |  |  |  |
| N0 | 12.50% (1/8) | 33.33% (3/9) | 0.576 |
| N1 | 50.00% (4/8) | 66.67% (6/9) | 0.637 |
| N2 | 25.00% (2/8) | 0.00% (0/9) | 0.206 |
| N3 | 12.50% (1/8) | 0.00% (0/9) | 0.471 |
| TNM stage |  |  |  |
| I | 12.50% (1/8) | 33.33% (3/9) | 0.576 |
| II | 50.00% (4/8) | 55.56% (5/9) | 1.000 |
| III | 37.50% (3/8) | 11.11% (1/9) | 0.294 |
| Tumor grading |  |  |  |
| G1 | 0.00% (0/8) | 11.11% (1/9) | 1.000 |
| G2-3 | 100.00% (8/8) | 88.89% (8/9) | 1.000 |

Acronyms: TNM = tumor nodes metastasis.

Supplemental Table 6- PITX2 (PMR) and immunoistochemical expression of TFEB, CARM1, SIRT1, and Beclin-1 divided into the two groups (cases who recurred within 12 months from treatment initiation and controls who did not recur).

|  | Recurrence within 12 months (8) | No recurrence (9) | p |
| --- | --- | --- | --- |
| PITX2 (PMR) | 4.50 (0.00-67.00) | 14.00 (7.00-17.00) | 0.661 |
| CARM1 (H-score) | 90.00 (56.88-191.25) | 16.25 (0.00-41.88) | <0.05 |
| SIRT1 (H-score) | 187.50 (172.50-196.25) | 98.75 (95.00-163.75) | <0.05 |
| TFEB (H-score) | 200.00 (196.00-200.00) | 90.00 (57.50-95.00) | <0.05 |
| Beclin-1 (H-score) | 180.00 (90.00-226.50) | 65.00 (12.00-70.00) | <0.05 |
